# Probing unnatural amino acid integration into enhanced green fluorescent protein by genetic code expansion with a high-throughput screening platform

**DOI:** 10.1186/s13036-016-0031-6

**Published:** 2016-09-30

**Authors:** Georg Wandrey, Joel Wurzel, Kyra Hoffmann, Tobias Ladner, Jochen Büchs, Lorenz Meinel, Tessa Lühmann

**Affiliations:** 1 AVT, Biochemical Engineering, RWTH Aachen University, Aachen, 52074 Germany; 2 Institute for Pharmacy and Food Chemistry, University of Würzburg, Am Hubland, Würzburg, 97074 Germany

**Keywords:** Amber codon suppression, Online monitoring system, High-throughput screening, Unnatural amino acid, Bio-orthogonal chemistry, Protein engineering

## Abstract

**Background:**

Genetic code expansion has developed into an elegant tool to incorporate unnatural amino acids (uAA) at predefined sites in the protein backbone in response to an amber codon. However, recombinant production and yield of uAA comprising proteins are challenged due to the additional translation machinery required for uAA incorporation.

**Results:**

We developed a microtiter plate-based high-throughput monitoring system (HTMS) to study and optimize uAA integration in the model protein enhanced green fluorescence protein (eGFP). Two uAA, propargyl-L-lysine (Plk) and (S)-2-amino-6-((2-azidoethoxy) carbonylamino) hexanoic acid (Alk), were incorporated at the same site into eGFP co-expressing the native PylRS/tRNA^Pyl^_CUA_ pair originating from *Methanosarcina barkeri* in *E. coli*. The site-specific uAA functionalization was confirmed by LC-MS/MS analysis. uAA-eGFP production and biomass growth in parallelized *E. coli* cultivations was correlated to (i) uAA concentration and the (ii) time of uAA addition to the expression medium as well as to induction parameters including the (iii) time and (iv) amount of IPTG supplementation. The online measurements of the HTMS were consolidated by end point-detection using standard enzyme-linked immunosorbent procedures.

**Conclusion:**

The developed HTMS is powerful tool for parallelized and rapid screening. In light of uAA integration, future applications may include parallelized screening of different PylRS/tRNA^Pyl^_CUA_ pairs as well as further optimization of culture conditions.

**Electronic supplementary material:**

The online version of this article (doi:10.1186/s13036-016-0031-6) contains supplementary material, which is available to authorized users.

## Background

The number of methods for site-specific protein modification, allowing precise conjugation of drugs or polymers e.g. for medical imaging or studying of biological processes with defined fluorescent probes, respectively, has substantially increased in recent years [1, 2]. Among them, amber codon suppression (genetic code expansion) has developed into an elegant tool to incorporate unnatural amino acids (uAA) at predefined sites in the protein backbone [3–5].

This method is based on the translation machinery evolved in archaebacteria, which are able to incorporate the 22^nd^ amino acid L-pyrrolysine (Pyl) as building block in proteins during translation beyond the typically used 20 canonical amino acids [6, 7]. Pyl is encoded by an amber termination codon (UAG) within a gene and is recognized by its orthogonal suppressor tRNA^Pyl^. The transfer of Pyl to its specific tRNA^Pyl^ is catalyzed by a specific pyrrolysyl-tRNA synthetase (PylRS). Due to high substrate side chain promiscuity of the PylRS enzyme, structural related Pyl analogues with distinct functional groups can be easily integrated to enable a broad chemical versatility for bioconjugation chemistry [8]. In fact up to 23 different functional uAA were shown to be incorporated using the native PylRS/tRNA^Pyl^_CUA_ pair originated from *Methanosarcina barkeri* (*M. Barkeri)* [9]*.* Moreover, numerous PylRS mutants have been engineered with improved activities and for recognition of Pyl derivatives, which are not targeted by the native PylRS enzyme. Whereas other orthogonal synthetase-tRNA pairs derived from strains like *Methanococcus jannaschii* are confined to bacterial cells without further genetic modifications, the genes of the PylRS-tRNA^Pyl^_CUA_ system from *Methanosarcina* species are of broad applicability and have been successfully transferred to incorporate uAA in proteins in more complex hosts such as yeast [10], mammalian cells [11] or multicellular organisms such as *Caenorhabditis elegans* [12]*.*

Protein yields, however, are usually lower compared to the expression levels of the wild-type analogue. Optimization of uAA incorporation using amber codon suppression includes (i) variation of uAA addition (concentration and time of addition to the expression medium), (ii) expression control for the PylRS/tRNA^Pyl^_CUA_ pair as well as for the gene of interest and (iii) quantification of the desired product. Up to now, reports are based on gel electrophoresis analysis and, therefore, rather qualitative or average yields of purified protein are reported [13, 14]. Furthermore, these do not allow online monitoring or high throughput screening of culture conditions as required for studies based on experimental design including the assessment of interactions among parameters (i.e. questions regarding the impact of one input parameter A depending on the level of another parameter B).

Consequently, we applied a high-throughput approach combined with online monitoring of microbial growth and product formation of a fluorescent reporter protein. Process parameters with relevance for the insertion of uAA by the PylRS-tRNA^Pyl^_CUA_ system in *E. coli* were identified and optimized. The system was further consolidated by end point-detection of the uAA modified fluorescent reporter protein in the bacterial supernatant using standard enzyme-linked immunosorbent procedures.

## Results and discussion

### Introduction of unnatural amino acids into eGFP by amber codon suppression

**Fig. 1:**
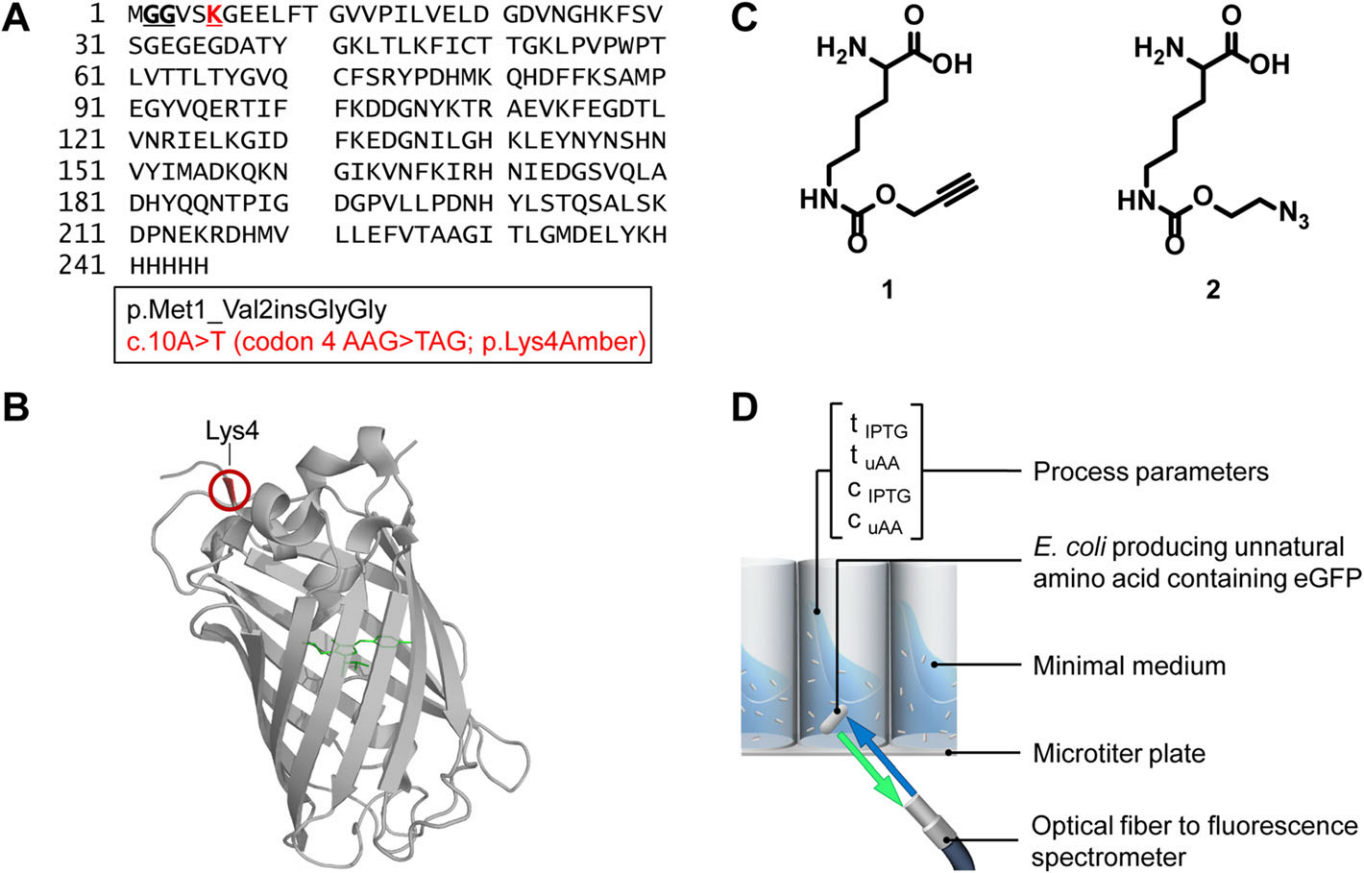
Introduction of unnatural amino acid into eGFP and high throughput screening. **a** The amino acid sequence of Lys-eGFP was extended with two Gly after position 1 and the unnatural amino acid was incorporated at the position of the amber stop codon TAG which was introduced at position 4 using (**b**) propargyl-L-lysine (Plk, **1**) or (S)-2-amino-6-((2-azidoethoxy)carbonylamino) hexanoic acid (Alk, **2**). **c** Beta barrel structure of eGFP (PDB ID: 2Y0G) with highlighted chromophore in the center and incorporation site for the unnatural amino acid at the N-terminus at position 4. **d** Non-invasive online monitoring of *E. coli* cultures, producing the unnatural amino acid containing eGFP. The process parameters unnatural amino acid concentration (c_uAA_), time of unnatural amino acid addition (t_uAA_), IPTG concentration (c_IPTG_) and time of IPTG addition (t_IPTG_) were studied in parallelized experiments

Enhanced green fluorescent protein (eGFP) was used to monitor uAA incorporation with the pylRS/tRNA^Pyl^_CUA_ pair originated from *M. barkeri* [14, 15]. The amber codon (UAG) was integrated into the eGFP sequence at the N-terminus (residue #4; Lys4/uAA) to exclusively monitor eGFP formation as result of the successfully integrated uAA through amber codon suppression (Fig. 1a). The /tRNA^Pyl^_CUA_ was constitutively expressed, whereas both the pylRS and the UAG-eGFP target gene were under lac operon control for induction with IPTG [14]. As substrates for the native PylRS/tRNA^Pyl^_CUA_ pair two different well-recognized Pyl derivatives were chosen: Plk (propargyl-L-lysine; 1) and Alk ((S)-2-amino-6-((2-azidoethoxy) carbonylamino) hexanoic acid; 2; Fig. 1b). The azide and alkyne functionalities of the selected uAA enable biorthogonal click chemistry as demonstrated by myoglobin [13], ubiquitin [14] or basic fibroblast growth factor [16] and for site-specific protein modification of the glycocalyx on living cells [17]. The formation of uAA-eGFP and biomass is monitored through the transparent bottom of microtiter plates with a screening platform constructed in-house in a modified BioLector setup [18, 19]. An optical fiber connected to a fluorescence spectrometer was positioned below the microtiter plates and allowed non-invasive online monitoring without interrupting the orbital shaking movement required for oxygen supply and mixing of the culture. The optical fiber automatically moved so quickly from well to well such that continuous monitoring of up to 4 microtiter plates was achieved providing on the fly comparison of various process parameters through quasi-simultaneous read-outs (Fig. 1d).

**Fig. 2:**
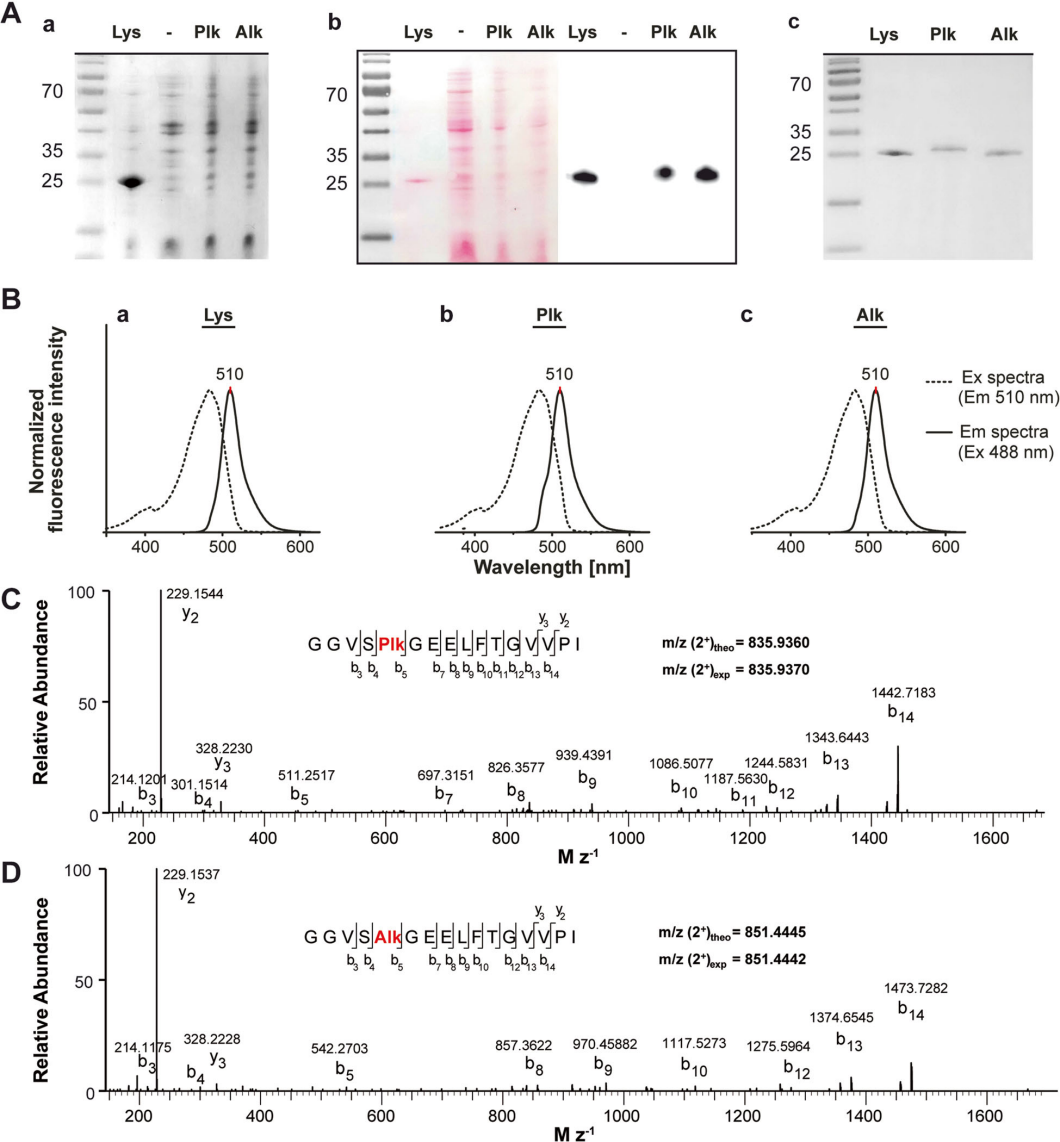
**a** Analysis of eGFP expression. *a* SDS-PAGE and *b* Western Blot and Ponceau S staining as loading control of total cell extracts. The positive control (Lys-eGFP) was diluted 1/10 for Western blot analysis to prevent overexposure. *c* SDS-PAGE of purified Plk-eGFP and Alk-eGFP in comparision to wild-typ Lys-eGFP. **b** normalized fluorescence spectra of Lys-eGFP, Plk-eGFP and Alk-eGFP. ESI-MS/MS spectra of elastase digested Plk-eGFP (**c**) and Alk-eGFP (**d**), respectively, with assigenment of the fragments to the uAA-eGFP amino acid sequence

Initially, we confirmed the successful incorporation of uAA into eGFP by amber codon suppression for two uAAs Plk-eGFP and Alk-eGFP (Fig. 1b; in parallel to expression of the control Lys-eGFP; Fig. 1c) using 3 mM uAA in TB-medium following standard expression procedures [15, 16]. Expression of Plk-eGFP and Alk-eGFP compared to Lys-eGFP (positive control) and to IPTG induced bacteria transformed with the pylRS/tRNAPylCUA pair but without the addition of the uAA (negative control) was analyzed in total cell lysates after 6 h of expression by SDS-PAGE (Fig. 2A,a) followed by Western blotting to confirm the protein’s identity (Fig. 2A,b). As expected expression of wild-type Lys-eGFP was highest as demonstrated by SDS-PAGE and Western blotting and in comparison to Plk-eGFP and Alk-eGFP, respectively. As next we isolated all eGFP constructs from cell lysates by metal ion affinity chromatography. Purification of all eGFP analogues resulted in high purity as determined by SDS-PAGE analysis (Fig. 2A,c). eGFP fluorescence is linked to proper eGFP folding into the characteristic GFP β-barrel structure (Fig. 1c). To investigate the effects of uAA insertion, which may interfere with the tertiary structure of eGFP, affecting its fluorescence properties, purified Plk-eGFP and Alk-eGFP were analyzed by fluorescence spectroscopy in comparison to the control protein (Fig. 2B). Both eGFP analogues showed identical fluorescence signatures with λ_max_ = 510 nm of eGFP as previously described [20], indicating that uAA insertion did not interfere with eGFP β-barrel maturation. MALDI-MS analysis suggested N-terminal Met removal from all eGFP constructs upon translation through *E. coli* derived methionine amino peptidase (MetAP) (Additional file 1: Figure S1). This finding was corroborated by elastase digests of Plk-eGFP and Alk-eGFP followed by LC-MS/MS analysis confirming the insertion of Plk (Fig. 2C) and Alk (Fig. 2D) at position #4 in the amino acid sequence. This characterized eGFP fluorescence reporter system marked the starting point for deploying the screening platform.

### Online measurement of biomass and Plk-eGFP formation

**Fig. 3:**
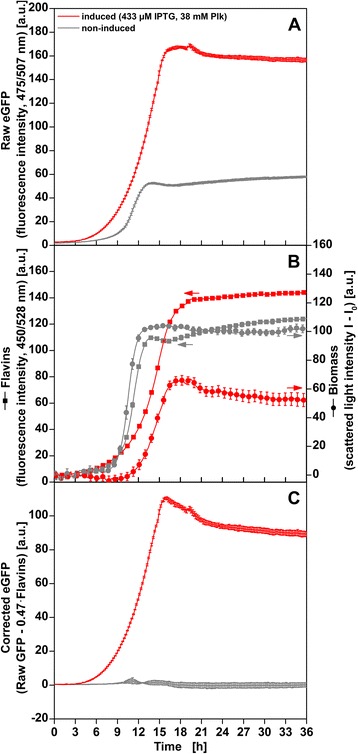
Correction for flavin autofluorescence. **a** Raw eGFP fluorescence intensity (λ_Ex_ = 475 nm, λ_Em_ = 507 nm) of non-induced cultures (grey) and induced cultures supplemented with 433 μM IPTG and 38 mM Plk (*red*). **b** Flavin fluorescence intensity (left axis, squares, λ_Ex_ = 450 nm, λ_Em_ = 528 nm) and scattered light intensity (right axis, circles, λ_Ex_ = 650 nm, λ_Em_ = 650 nm). Only every 5^th^ data point is shown to improve readability. **c** Corrected eGFP signal (I_eGFP,corrected_ = I_eGFP,raw_ – 0.47 I_flavin_) as applied in subsequent figures. Error bars indicate standard deviation of triplicates

During initial cultivations an increase in raw eGFP fluorescence intensity (475/507 nm) was not exclusively detected for induced cultures expressing Plk-eGFP but also for non-induced cultures with about 40 % final fluorescence intensity compared to induced cultures (Fig. 3a). ELISA measurements confirmed the formation of Plk-eGFP in induced cultures but the absence of eGFP in non-induced cultures (data not shown). We hypothesized that a superimposition of biogenic flavin fluorescence was the cause for the detected signal increase in non-induced cultures. In contrast to commercially available BioLector setups that rely on fixed combinations of excitation and emission filters for detection of fluorescence signals the monochromators of the in-house constructed HTMS enable measurements at all wavelength combinations in the UV-Vis range. With this system in our hands, we selectively monitored in parallel an additional flavin fluorescence intensity signal over the course of the cultivation (450/528 nm, Fig. 3b, left axis, *squares*). For non-induced cultures (*grey*), this signal correlated well to the scattered light intensity indicating biomass formation (650/650 nm, right axis, *circles*). Induced cultures (*red*) showed an over proportional increase driven by the overlapping fluorescence of the formed Plk-eGFP. By applying routinely used unmixing methods the eGFP fluorescence signal could be corrected by subtracting the flavin signal multiplied by the ratio of raw eGFP to flavin fluorescence intensity at the end of non-induced cultivations after 36 h (I_eGFP,corrected_ = I_eGFP,raw_ – 0.47 I_flavin_) [21, 22]. As expected the corrected eGFP signal showed a strong increase during cultivation of induced cultures and alternated around zero for non-induced cultures (Fig. 3c). All following eGFP signals were corrected for autofluorescence applying this method.

**Fig. 4:**
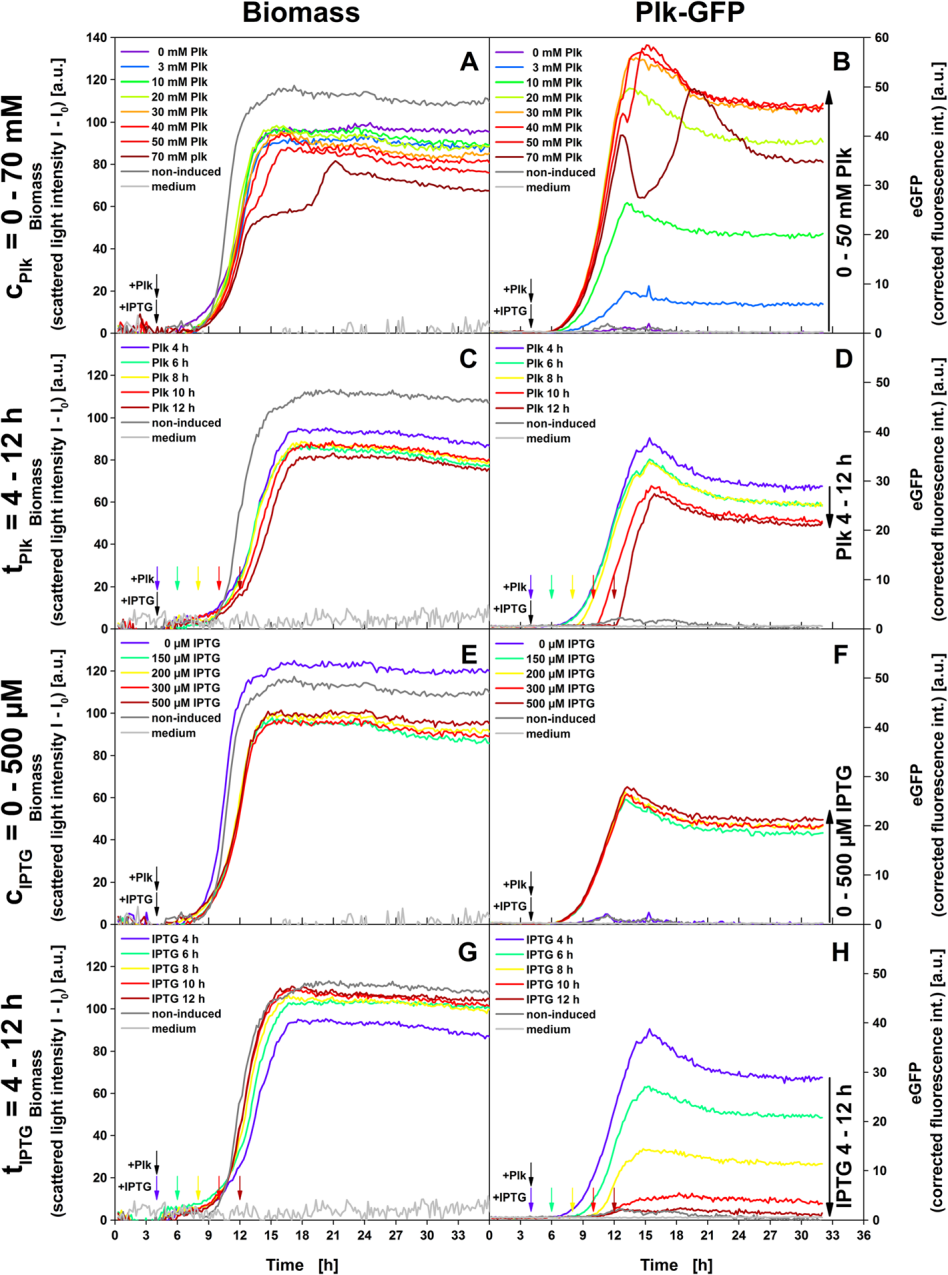
Online measurement of biomass and Plk-eGFP formation. Scattered light intensity as a measure for biomass (first column) and eGFP fluorescence intensity (second column) over time of culture of *E. coli* BL21(DE3) uAA-eGFP cultures. Four process parameters were varied individually: (**a**, **b**) Plk concentration c_plk_ = 0–70 mM; (**c**, **d**) time of Plk addition t_plk_ = 4–2 h (**e**, **f**) IPTG concentration c_IPTG_ = 0–500 μM (**g**, **h**) and time of IPTG addition t_IPTG_ = 4 – 12 h. If not varied, parameters were fixed at c_Plk_ = 10 mM_,_ t_Plk_ = 4 h, c_IPTG_ = 200 μM and t_IPTG_ = 4 h respectively. No Plk and no IPTG was added to cultures designated as ‘non-induced’ (dark grey). Small down-pointing arrows indicate the time of Plk and IPTG supplementation and large vertical arrows indicate trends in eGFP fluorescence after 32 h

Four process parameters were screened in parallel for relevance to uAA incorporation: (i) the uAA concentration (c_uAA_) and (ii) time of supplementation (t_uAA_) as well as (iii) the inducer concentration (c_IPTG_) and (iv) the time of induction (t_IPTG_) (Fig. 1d). These parameters were systematically varied in parallelized cultivations and biomasses as well as Plk-eGFP formation were monitored online. The online monitoring experiments indicated a minor impact of the Plk concentration on biomass formation (Fig. 4a). Up until the end of the exponential growth phase after 12 h the scattered light signals quantitatively reflecting biomass concentrations were very similar for 0–70 mM Plk. After 12 h cultures with high Plk concentrations (50 mM, 70 mM) showed lower growth rates resulting in 14–24 % lower biomass at the end of the experiment. In contrast, Plk-eGFP formation strongly depended on Plk concentration (Fig. 4b) peaking at the end of the growth phase after approx. 15 h. Increasing the Plk concentration from 0–30 mM strongly increased Plk-eGFP formation, while further supplementation up to 50 mM Plk did not impact outcome. The observed drop in Plk-eGFP formation at 70 mM possibly reflected growth limitations as indicated in the biomass signal (Fig. 4a).

The time of Plk supplementation did not impact biomass formation (Fig. 4c). In contrast, eGFP fluorescence was detected within 30 min after Plk supplementation (Fig. 4d). Especially late Plk supplementations after 10 or 12 h of cultivation – when more biomass was present for eGFP formation – sparked a steep increase in eGFP fluorescence. The fast fluorescence response measured with the online monitoring system provided evidence that the PylRS catalyzed transfer of Plk to the tRNA was not rate limiting under the selected culture conditions and sufficient expression level of PylRS were achieved. Later Plk supplementation decreased eGFP fluorescence signals as compared to earlier time-points demonstrating the positive impact of early Plk supplementation on Plk-eGFP formation.

Induction with IPTG decelerated growth (Fig. 4e). Supplementation with 150–500 μM IPTG retarded the end of the exponential phase by 1.5 h as compared to non-induced cultures and decreased final scattered light intensity by about 20 % (Fig. 4e). These reductions in biomass following IPTG reflect the metabolic burden introduced by the heterologous protein production [23]. The amount of formed eGFP was not impacted by c_IPTG_ as tested within a range of 150–500 μM (Fig. 4f). t_IPTG_ scarcely impacted biomass formation (Fig. 4g). Early induction after 4 or 6 h slightly reduced growth rates reflecting the shift of cellular resources from biomass formation into eGFP formation (Fig. 4g). Consequently, earlier t_IPTG_ resulted in higher eGFP formation (Fig. 4h).

Online fluorescence monitoring also showed a delay of at least 1 h between IPTG supplementation and eGFP detection (Fig. 4h). This is in contrast to shorter response time of about 30 min following Plk addition (Fig. 4d). This difference at least partly reflects the additional time requirement for the assembly of PylRS and tRNA^Plk^_CUA_ as a prerequisite for Plk-eGFP expression.

### Optimization of uAA incorporation and induction parameters

**Fig. 5:**
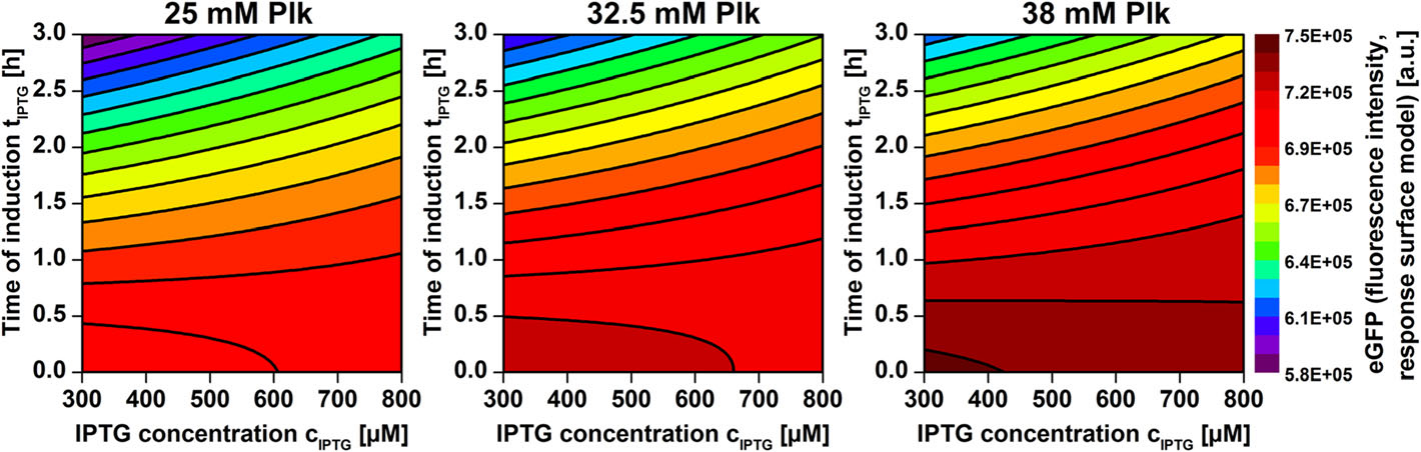
Response surface model for Plk-eGFP production. eGFP fluorescence of *E. coli* BL21(DE3) uAA-eGFP cultures after 24 h as predicted by the response surface model as a function of Plk concentration, IPTG concentration and time of IPTG addition (t_plk_ = 0 h). Dark *red* colors indicate operating conditions which lead to high Plk-eGFP fluorescence

We now shifted the experimental design (one parameter was varied while the other three were constant) to a two-stage screening in full-factorial design mode for rigorous parameter optimization. The first stage was used to identify the most significant parameters and to shift and narrow the design space around the optimum (Additional file 1: Table S1). In the second stage a response surface model including quadratic interactions was constructed as linear combination of significant terms (main: c_Plk_, c_IPTG_, t_IPTG_; linear interaction: c_IPTG_^.^t_IPTG_; quadratic interaction: t_IPTG_^2^, Additional file 1: Figure S2). The model predicted the Plk-eGFP fluorescence intensity (I_Plk-eGFP_, [a.u.]) as a function of Plk concentration (c_Plk_, [mM]), IPTG concentration (c_IPTG_, [μM]) and the time of IPTG addition (t_IPTG_, [h]) following I_Plk ‐ eGFP_ = 2489 ⋅ c_Plk_ ‐ 27.02 ⋅ c_IPTG_ ‐ 27561 ⋅ t_IPTG_ ‐ 9456 ⋅ t_IPTG_^2^ + 42.02 ⋅ c_IPTG_ ⋅ t_IPTG_ + 696949, adequately describing the system (Additional file 1: Figure S3). This result was confirmed by an independent validation run (Additional file 1: Figure S4) and was graphically represented (Fig. 5). As indicated by dark red colors, highest eGFP formation followed 300–400 μM IPTG when added at the beginning of the experiment (0–0.5 h of cultivation) and when high Plk concentrations (38 mM) were added right at the start of the cultivation. The commonly applied method of IPTG induction in the early exponential growth phase (after approx. 9 h) was, therefore, inappropriate to yield the optimum of Plk-eGFP formation within the specific pattern described herein [24].

**Fig. 6:**
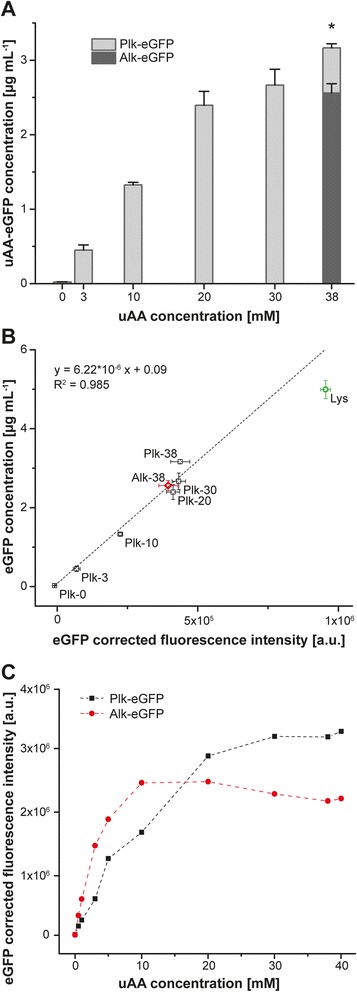
**a** Concentration of uAA-eGFP after 24 h in culture as a function of uAA concentration. Each column represents the mean + SD of four independent experiments (*p* < 0.001). **b** Comparison between protein concentrations of eGFP analogues as determined by ELISA and online measured fluorescence intensities of eGFP analogues. Data points illustrated as squares and labeled with Plk represent Plk-eGFP samples, whereas diamonds and circles display Alk-eGFP and Lys-eGFP, respectively. Tags indicate the uAA concentration in mM. All results are given as mean ± SD (*n* = 4) (**c**). Online measured fluorescence intensities of eGFP analogues in respect to uAA concentration. uAA and IPTG (433 μM) were added at the start of the cultivation

IPTG concentrations of 400–1000 μM IPTG yielded comparable outcome reflecting saturated protein formation capacities and opening the possibility of reducing IPTG while maintaining maximum Plk-eGFP formation (Additional file 1: Figure S5). The set point aiming for a robust process outcome (i.e. deviation of input parameters from the set point would not significantly impact the Plk-eGFP concentration) were calculated via Monte-Carlo simulation at c_Plk_ = 38 mM, t_Plk_ = 0 h, c_IPTG_ = 433 μM, t_IPTG_ = 0.2 h (Additional file 1: Figure S6) and characterized by measuring Plk-eGFP concentrations using ELISA. Corroborating the online fluorescence measurements (Fig. 3), Plk-eGFP concentrations strongly depended on the Plk concentration (Fig. 6a). With 38 mM Plk and 433 μM IPTG added at the beginning of the cultivation a Plk-eGFP concentration of 3.17 ± 0.06 μg mL^-1^ was achieved while with a generally used concentration of 3 mM Plk only 0.45 ± 0.07 μg mL^-1^ were produced under otherwise identical experimental conditions. eGFP concentrations (determined by ELISA) and eGFP fluorescence intensities detailed by the HTMS correlated linearly (Fig. 6b).

Side chain promiscuity of the PylRS enzyme allows the incorporation of different uAAs than Plk into eGFP using the same setup. Instead of Plk’s terminal alkyne functionality we now changed to an azide functionality Alk ((S)-2-amino-6-((2-azidoethoxy) carbonylamino) hexanoic acid) (Fig. 1b). Both, terminal azide and alkyne functionalities profile proteins for copper(I)-catalyzed azide alkyne cycloaddition (CuAAC) [25, 26]. Straightforward extrapolating of the optimized set point found for Plk-eGFP formation to Alk-eGFP resulted in immediately achieved concentrations of 2.56 ± 0.13 μg mL^-1^ (Fig. 6a, black bar; Fig. 6b, Alk-38). The amount of supplied Alk critically drove uAA-eGFP formation as read from online monitored fluorescence (Fig. 6c). Starting off these results, rapid process optimization for Alk by using 10 mM Alk instead of 30 mM Plk readily boosted maximum eGFP fluorescence intensities beyond the results obtained for Plk-eGFP, possibly reflecting a higher PylRS affinity for Alk in comparison to Plk. Nevertheless, both concentrations found for the optima (10 mM for Alk and 30 mM for Plk) were far beyond commonly applied uAA concentration, typically in the range of 1 mM [27–30], although higher uAA concentrations have already been linked to increased target protein concentration as estimated after expression and purification [31–33]. However, high uAA concentrations – as demonstrated for Alk and Plk here – are not cost-effective for large scale expression and are therefore still the limiting factor in genetic code expansion technology.

The HTMS allows for rapid screening and the examples provided above demonstrated the power of parallelized assessments, yielding rapid optimization of culture conditions while readily balancing the need for high titers and robust processes for reproducible batch-to-batch outcome. Optima identified for Plk-eGFP and Alk-eGFP concentrations approximated titers of wild-type, unmodified Lys-eGFP (4.98 ± 0.23 μg mL^-1^ produced under the same conditions (Fig. 6b, Lys) performing at 36 % and 49 % relative to the wild-type, respectively. Recent progress in the field of genetic code expansion can conveniently be screened and optimized by this setup. This includes the selection of improved uAA tRNA synthetases [28, 31, 34], the use of special ribosomes for uAA incorporation and systems addressing the competition of the uAA-tRNA with release factor 1 (RF1) at the amber stop codon [35–37]. The system can also serve by providing a more holistic landscape rather than point measurements for mechanistic studies in analogy to investigations into the role of tRNA delivery to the ribosome [38, 39].

Obviously, each protein requires the identification of a new design space and uAA-eGFP was herein used to demonstrate the proof of concept. For uAA-GFP, a very early induction optimum (t_IPTG_ = 0.2 h) was determined with the two-stage screening. Induction after just 3 h of cultivation already impaired target protein production by 15 % (Fig. 5). Furthermore, no deviations in growth rates were detected upon addition of up to 40 mM of Plk (Fig. 4a). This shows that unintended uAA incorporation into host protein that could reduce fitness and product formation capabilities is of no concern here. If that was the case, the optimal induction time would lie after the early exponential growth phase when further cell growth has a lesser effect on final product concentration. The early induction optimum also reflects the general need for early induction times when working within the specific context of uAA incorporation, caused by the additional requirement of tRNA^uAA^_CUA_ pair formation as compared to wild type expression. Previous studies already demonstrated the advantages of independently controlling the incorporation machinery versus the target gene, thereby fine-tuning protein ratios for uAA incorporation [32]. Moreover, the influence of the location of the amber codon within the gene – ranging from initial to terminal integration of the uAA at the ribosome – on eGFP expression levels can be detailed by the HTMS presented here.

High-throughput experimentation with concise optimization steps is highly beneficial to quickly adjust parameters for each target protein. Current studies starting off what is described here aim at expanding the application to non-fluorescent proteins. While the overall approach remains identical, these systems need to shift from monitoring of fluorescent proteins (Fig. 1d) to other available online signals like biomass and oxygen transfer rate. Preliminary experiments already indicated that a measurement of the metabolic burden induced by heterologous protein formation can be used to assess non-fluorescent protein formation during genetic code expansion.

## Conclusions

We provide a high throughput assessment platform designed for parallel screening and optimization studies. The platform allows massive data generation leading to optimized design spaces for input parameters, including but not limited to the use of novel uAA, advanced expression systems, future synthetases, location of the amber codon and multiple uAA integration sites or allows in-depth mechanistic studies with high comparability and reliability.

## Methods

### Materials

(S)-2-amino-6-((2-azidoethoxy)carbonylamino)hexanoic acid (Alk) was purchased from IRIS Biotech GmbH (Marktredwitz, Germany) or was kindly provided by EMC Microcollections GmbH (Tübingen, Germany). Restriction endonucleases were from New England Biolabs (Ipswitch, MA). Pfu DNA polymerase was from Stratagene (La Jolla, CA). Boc-protected L-lysine was from P3 BioSystems LLC (Shelbyville, KY). Coomassie Brilliant Blue G250 and Bradford Protein Assay Kit were from Pierce (Rockford, USA). GFP ELISA Kit Simple step (#ab171581) was from Abcam (Cambridge, United Kingdom). Acetonitrile (HPLC grade) and trifluoroacetic acid (HPLC grade) were from VWR (Ismaning, Germany). Anti-GFP Antibody #2555 and Anti-rabbit IGG HRP-linked Antibody # 7074 were purchased from Cell Signaling (Hitchin, United Kingdom). Super Signal West Pico Luminescent Substrate was purchased from Thermo Scientific (Waltham, MA). All other chemicals used were at least of pharmaceutical grade and were purchased from Sigma-Aldrich (unless noted otherwise). Propargyl-L-lysine (Plk) was prepared as HCl-salt as previously described [16].

### Subcloning, expression and purification of eGFP analogues (Lys-eGFP, Plk-eGFP and Alk-eGFP)

The pEGFP-N1 plasmid containing the gene encoding for full length enhanced green fluorescent protein (eGFP) were from Clontech Laboratories, Inc. (Mountain View, CA). For the Lys-eGFP mutant, the initial cDNA was amplified by PCR using a forward primer including an NdeI restriction site and two additional glycine codons inserted after the methionine start codon (5‘-CCCCATATGGGCGGTGTGAGCAAGGGCGAGGAGCTG-3’), while deploying a reverse primer including a 6 × histidine tag and an EcoRI restriction site (5‘CCCGGATCCTTAGTGGTGATGGTGATGATGCTTGTACAGCTCGTCCATGCCG-3’). In order to obtain 4(TAG)-eGFP, the forward primer was altered to 5‘CCCCATATGGGCGGTGTGAGCTAGGGCGAGGAGCTG-3’ substituting AAG (Lys) with amber codon TAG. After digestion with NdeI and EcoRI, the resulting cDNAs Lys-eGFP and 4(TAG)-eGFP were subcloned into the backbone of a pET11a vector-construct, containing the gene for the pyrrolysine tRNA, the lipoprotein promotor lpp, the terminator RRN b/c and an ampicillin resistance gene as described in Eger et al. [14] By means of T7-term promoter based DNA sequencing the correct sequence of both inserts was confirmed. Subsequently, the pET11a plasmids were co-transformed with a pRSF-duet vector, providing the gene for the pyrrolysine tRNA synthetase and kanamycin resistance, into the *E. coli* BL21(DE3) for amber codon suppression as previously described [25].

For SDS-page, fluorescence spectra and MALDI-MS, bacteria were cultured in 2 L baffled flasks inoculated with 1 % overnight culture at 37 °C and 130 rpm in 500 mL Terrific Broth (TB) medium supplemented with 100 μg mL^-1^ carbenicillin, 34 μg mL^-1^ kanamycin, 2 mM MgSO_4_ and 50 μL of polypropylene glycol as an anti-foam agent in an environmental Shaker 10× 400 (SANYO Gallenkamp, Leicestershire, UK) with a shaking diameter of 32 mm [15, 16]. Plk or Alk were added to a final concentration of 3 mM at an OD_600_ = 0.4. eGFP expression was induced with 1000 μM IPTG at OD_600_ = 0.6-0.7 and the bacteria were cultivated at 33 °C and 130 rpm. After 6 h, the bacteria were harvested by centrifugation and the pellets were washed and resuspended in lysis buffer (20 mM phosphates, 500 mM NaCl and 25 mM imidazole, pH 7.5). Cells were solubilized by cell disruption with a SONOPULS Ultrasonic homogenizer HD 3100 system (Bandelin, Berlin, Germany) at 4 °C, in lysis buffer containing 1 mM PMSF. After centrifugation at 100.000 g for 1 h at 4 °C (L8-60 M Ultracentrifuge, Beckman-Coulter, Brea, CA), the supernatant with His-tagged eGFP mutant was purified by immobilized metal ion affinity chromatography deploying an FPLC system (Aekta Purifier, GE, Freiburg, Germany) with a HisTrap FF (Ni Sepharose) crude 1 mL column (GE, Freiburg, Germany). Elution was initiated using a linear gradient of imidazole ranging from 25 mM to 500 mM. Combined fractions were dialyzed against PBS and the concentrations were determined by Bradford protein assay following the manufacturer’s instructions. Lys-eGFP was isolated in average yields of 15 μg mL^-1^ expression culture and the average yield of Plk-eGFP and Alk-eGFP was approximately 2 μg mL^-1^ expression culture, respectively.

### Cell lysate analysis and western blotting

1 mL bacterial suspension of each expression was centrifuged and gently washed in PBS. Obtained pellets were resuspended in SDS-loading buffer, lysed at 95 °C and centrifuged again. Sample supernatants were then analyzed by SDS-PAGE followed by Western blotting. The supernatant of the positive control (Lys-eGFP) was diluted 1:10 for Western blot analysis. As loading control, the blotted nitrocellulose membrane was stained with Ponceau S (0.2 % solution in water) prior before incubation with an anti-GFP antibody (1:1000 in Tris-buffered saline, containing 0.1 % (w/w) Tween 20). After incubation with a peroxidase conjugated secondary antibody (1:1000 in Tris-buffered saline, containing 0.1 % (w/w) Tween 20), the signal intensity was assessed using a Super Signal West Pico Luminescent Substrate and a FluorChem FC2 imaging system from Protein Simple (Santa Clara, CA).

### SDS-PAGE

Expressed proteins were analyzed by standard tris-glycine SDS-polyacrylamide gel electrophoresis. Gels were stained with Coomassie Brilliant Blue G250 and photographed using a FluorChem FC2 imaging system (ProteinSimple, San Jose, CA).

### Fluorescence spectroscopy

Fluorescence spectra were obtained on a LS 50B Fluorescence Spectrometer (PerkinElmer, Waltham, MA). All spectra scans were recorded from 200–800 nm with a scan speed of 150 nm min^-1^, applying a solution of each eGFP analogue with a concentration of 30 μg mL^-1^ in a quartz cuvette as measuring cell. Emission spectra were excited at 488 nm and excitation spectra where monitored at 510 nm following the predefined values reported in [20]. The slit width was set to 4.7 nm for emission and 9.7 nm for excitation.

### MALDI-MS

A solution of 20 μg in 50 μL of protein sample was acidified with 0.1 % TFA and desalted using Zip Tip® pipette tips (C18 resin, Millipore, Billerica, USA) according to the manufacturer’s instructions. One μL of the eluate was embedded in a matrix, consisting of equal parts of 4-Bromo-α-cyanocinnamic acid and ACN/0.1 % TFA in water (1:4). Matrix-assisted laser desorption ionization (MALDI)-MS spectra were acquired in linear positive mode with a 337 nm wavelength nitrogen laser (Autoflex II LRF, Bruker Daltonics Inc., Billerica, USA). Mass spectra were calibrated externally with protein standard I (Bruker Daltonics Inc., Billerica, USA) containing insulin, ubiquitin, myoglobin and cytochrome C. Theoretical masses of wild-type proteins were calculated (http://web.expasy.org/peptide_mass) and adjusted for theoretical masses of non-canonical amino acids if necessary.

### NanoLC MS/MS

For in-gel digestion excised gel bands were destained with 30 % ACN, shrunk with 100 % ACN, and dried in a Vacuum Concentrator (Concentrator 5301, Eppendorf, Hamburg, Germany). Digests with elastase was performed overnight at 37 °C in 0.1 M NH_4_HCO_3_ (pH 8). About 0.1 μg of protease was used for one gel band. Peptides were extracted from the gel slices with 5 % formic acid. NanoLC-MS/MS analyses were performed on an LTQ-Orbitrap Velos Pro (Thermo Scientific) equipped with an EASY-Spray Ion Source and coupled to an EASY-nLC 1000 (Thermo Scientific). Peptides were loaded on a trapping column (2 cm × 75 μm ID. PepMap C18 3 μm particles, 100 Å pore size) and separated on an EASY-Spray column (25 cm × 75 μm ID, PepMap C18 2 μm particles, 100 Å pore size) with a 30 min linear gradient from 3–30 % ACN and 0.1 % formic acid. MS scans were acquired in the Orbitrap analyzer with a resolution of 30,000 at m z^-1^ 400 for MS scans and 7,500 at m z^-1^ 400 for MS/MS scans using HCD fragmentation with 30 % normalized collision energy. A TOP5 data-dependent MS/MS method was used; dynamic exclusion was applied with a repeat count of 1 and exclusion duration of 30 s; singly charged precursors were excluded from selection. Minimum signal threshold for precursor selection was set to 50,000. Predictive AGC was used with a target value of 1e6 for MS scans and 5e4 for MS/MS scans. Lock mass option was applied for internal calibration in all runs using background ions from protonated decamethylcyclopentasiloxane (m z^-1^ 371.10124).

### Cultivation conditions

A two stage precultivation was performed in 250 mL shake flask on orbital shakers (LS-X, Kuhner, Switzerland) with a filling volume of 10 mL, a shaking frequency of 350 rpm, a shaking diameter of 50 mm and an initial OD_600_ of 0.1 for all cultivation stages [23]. The first precultivation stage was inoculated from cryogenically preserved cultures and conducted at 37 °C in Terrific Broth (TB) medium (5 g L^-1^ glycerol, 24 g L^-1^ yeast extract, 12 g L^-1^ tryptone, 12.54 g L^-1^ K_2_HPO_4_, 2.3 g L^-1^ KH_2_PO_4_; all medium components from Roth, Germany) [40]. After 4 h of cultivation, the first precultivation stage was used to inoculate the second precultivation stage which was performed at 30 °C in modified Wilms-MOPS minimal (WM) medium (20 g L^-1^ glucose, 6.98 g L^-1^ (NH_4_)_2_SO_4_, 3 g L^-1^ K_2_HPO_4_, 2 g L^-1^ Na_2_SO_4_, 41.85 g L^-1^ (N-morpholino)-propanesulfonic acid (MOPS), 0.5 g L^-1^ MgSO_4_ · 7H_2_O, 0.01 g L^-1^ thiamine hydrochloride, 1 mL L^-1^ trace element solution [0.54 g L^-1^ ZnSO_4_ · 7H_2_O, 0.48 g L^-1^ CuSO_4_ · 5H_2_O, 0.3 g L^-1^ MnSO_4_ · H_2_O, 0.54 g L^-1^ CoCl_2_ · 6H_2_O, 41.76 g L^-1^ FeCl_3_ · 6H_2_O, 1.98 g L^-1^ CaCl_2_ · 2H_2_O, 33.4 g L^-1^ Na_2_EDTA (Titriplex III)], pH adjusted to 7.5 with NaOH) [41]. After 7 h of cultivation, this second preculture stage was used to inoculate the main culture in 48-well FlowerPlates (MTP-48-B, lot 15xx, m2p-labs, Germany) which was performed at 30 °C with WM medium. A filling volume of 780 μL per well, a shaking frequency of 1000 rpm and a shaking diameter of 3 mm were used. The plates were sealed with a sterile self-adhesive polyolefin sealing foil (900371, HJ-Bioanalytik, Germany) to reduce evaporation while still allowing sufficient gas transfer. During cultivation, final concentrations of IPTG (0–1000 μM) and uAA (0–80 mM) were adjusted by adding 20–70 μL of concentrated stock solutions after shortly reducing the shaking frequency to 100 rpm. All media were supplemented with 100 μg mL^-1^ carbenicillin and 34 μg mL^-1^ kanamycin.

### Online monitoring of uAA-eGFP formation and biomass growth (BioLector)

Scattered light and fluorescence measurements were performed through the transparent bottom of the microtiter plates with an in-house constructed screening system based on the established BioLector setup [18, 19, 42]. In short, a quartz/quartz multi-mode fiber (LUV 105 μm, LEONI, Germany) was moved sequentially below the wells of up to four microtiter plates by a Cartesian motion system (CMS, Bosch Rexroth, Germany). The fiber was connected to a spectrofluorometer with excitation/emission monochromators (Fluoromax-4, HORIBA Jobin Yvon GmbH, Germany) and allowed quasi-continuous and contactless measurements on up to 4 microtiter plates in parallel without stopping the shaking movement which otherwise might have resulted in cell sedimentation and oxygen limitation.

For each well eGFP fluorescence intensity (I_eGFP,raw_) was measured for 600 ms at an excitation wavelength of 475 nm, an emission wavelength of 507 nm and a bandpass of 6 nm. Flavin fluorescence intensity (I_flavin_) were measured for 600 ms at an excitation wavelength of 450 nm, an emission wavelength of 528 nm and a bandpass of 6 nm to correct the eGFP signal for biogenic autofluorescence (I_eGFP,corrected_ = I_eGFP,raw_ – 0.47 I_flavin_). Backscatter intensity as a signal for biomass was monitored for 900 ms at 650 nm with a bandpass of 4 nm. Correlations between backscatter intensity, optical density and cell dry weight can be established as described previously [19, 43]. For raw eGFP and flavin fluorescence measurements the mean relative standard deviations of two sets of triplicates (non-induced, induced) over a cultivation time of 36 h (326 data points) were 1.15 ± 0.63 % and 0.95 ± 0.72 % respectively (Fig. 3). Relative standard deviation of corrected end-point eGFP fluorescence as applied in the screening stage was 2.9 % (*n* = 4).

### Response surface model

The formation of Plk-eGFP was analyzed as a function of four process parameters: (i) Plk conc., (ii) Plk time of addition, (iii) IPTG conc. and (iv) IPTG time of addition. The results of the first screening stage which considered the four process parameters (main effects) and linear interactions (Additional file 1: Table S1) were used to estimate the design space for the second stage which additionally considered quadratic interactions in central composite face-centered design (Additional file 1: Figure S2). The model coefficients were scaled and centered to allow a comparison of effects and their significance was determined (Additional file 1: Figure S3). The significant terms were used to construct a response surface model that predicts Plk-eGFP fluorescence as a function of Plk conc., IPTG conc. and IPTG time of addition. A validation run with 12 conditions plus center point was performed and showed that predicted and measured fluorescence intensities were in good agreement (Additional file 1: Figure S4). A robust setpoint for Plk-eGFP formation was determined by Monte-Carlo simulation (50,000 predicted fluorescence intensities, minimum threshold: 710,000 a.u., median: 739,511 a.u., Additional file 1: Figure S6). Experiments were conducted in duplicates (triplicate for center points). Design of experiments and data analysis were performed with MATLAB (R2012b, The MathWorks, USA) and MODDE Pro (v11.0.0.1717, MKS Umetrics AB, Umeå, Sweden) and in accordance with the manufacturer’s instructions. The amount of Plk-eGFP produced at the optimized set points was subsequently determined by ELISA.

### eGFP quantification by Enzyme Linked Immunosorbent Assay (ELISA)

Precultivations for ELISA measurements were performed as described for the online monitoring experiments. The main cultivation was conducted in shake flasks instead of microtiter plates to generate sufficient biomass for subsequent analysis. Cultivation conditions were the same as in the second precultivation step described above (V_L_ = 10 mL, n = 350 rpm, d_0_ = 350 rpm, T = 30 °C). The production of uAA-eGFP was induced with a final IPTG concentration of 433 μM and 0–38 mM uAA at the start of the cultivation. After 24 h of cultivation, eGFP fluorescence was measured in 48-well FlowerPlates as described for the online monitoring experiments. Additionally, bacterial pellets from 8 mL expression cultures were washed in PBS and resuspended in 1 mL extraction buffer supplemented with extraction enhancer solution provided by the eGFP ELISA Kit resulting in a total volume of approximately 1.2 mL bacterial suspension. 1 mL of this suspension was transferred into a 2 mL tube and 10 μL of a 0.1 M PMSF solution as well as 0.5 μL poly(propylene) glycol were added. Cell lysis was performed with a SONOPULS Ultrasonic homogenizer HD 3100 system in six sonication cycles (Bandelin, Berlin, Germany). Each cycle lasted for 30 s applying 0.6 s pulses in 1.2 s intervals with an amplitude of 80 % followed by a pause of 45 s. After this procedure samples were centrifuged at 12.000 g for 20 min at 4 °C and aliquots of the supernatants were used for eGFP quantification. ELISA was performed following the manufacturer’s instructions. Absorbance of the acidified 3,3’,5,5’-tetramethylbenzidine diimine product was determined at 450 nm using a Spectramax 250 microplate reader (Molecular Devices, Sunnyvale, CA). All preparation steps were conducted on ice and with precooled solutions. Data were analyzed by a Welch’s *t*-test using Minitab 17 (Minitab, Coventry, UK). Presented data are depicted as mean + SD; results were considered statistically significant at *p* ≤ 0.001.

## Additional file

Additional file 1: Supplemental **Figures S1**–**S7**, **Table S1** and further information are given in the SI. (PDF 455 kb)

## Abbreviations

Alk: (S)-2-amino-6-((2-azidoethoxy)carbonylamino)hexanoic acid
eGFP: enhanced green fluorescent protein
ELISA: enzyme-linked immunosorbent assay
HTMS: high-throughput monitoring system
Plk: propargyl-L-lysine
Pyl: L-pyrrolysine
PylRS: pyrrolysyl-tRNA synthetase
uAA: unnatural amino acids
