# Supplementary material for: Probing unnatural amino acid integration into enhanced green fluorescent protein by genetic code expansion with a high-throughput screening platform
Source: J Biol Eng. 2016 Sep 30;10:11. doi: 10.1186/s13036-016-0031-6 (PMC5045631; doi:10.1186/s13036-016-0031-6)
Supplement: Additional file 1: — Supplemental Figures S1–S7, Table S1 and further information are given in the SI. (PDF 455 kb) [file 13036_2016_31_MOESM1_ESM.pdf]

Supplemental Information

Probing unnatural amino acid integration into enhanced green fluorescent protein by genetic code expansion with a high-throughput screening platform

Georg Wandrey[1#], Joel Wurzel[2#], Kyra Hoffmann[1], Tobias Ladner[1], Jochen Büchs[1], Lorenz Meinel[2], Tessa Lühmann[2,*]

[1]AVT, Biochemical Engineering, RWTH Aachen University, DE-52074 Aachen, Germany

[2]Institute for Pharmacy and Food Chemistry, University of Würzburg, Am Hubland, DE-97074 Würzburg, Germany

Figures: S1–S7

Table: S1

Pages: 1– 11

Table S1. Range of process parameters. Four process parameters were studied for their impact on Plk-eGFP production in a two-step modeling approach. The parameter range used for the screening stage (first step) was estimated from preliminary experiments (**Figure 5**). The parameter range for the response surface model (second stage) was then determined in the screening stage (**Figure S2**).

**Process parameter**	**Symbol**	**Unit**	**Range (screening)**	**Range (response surface model)**
**Plk concentration**	c_{plk}	mM	20 – 40	25 – 40
**Plk time of addition**	t_{plk}	h	2 – 6	*fixed to 0 h*
**IPTG concentration**	c_{IPTG}	µM	50 – 1000	300 – 800
**IPTG time of addition**	t_{IPTG}	h	2 – 6	0 – 3

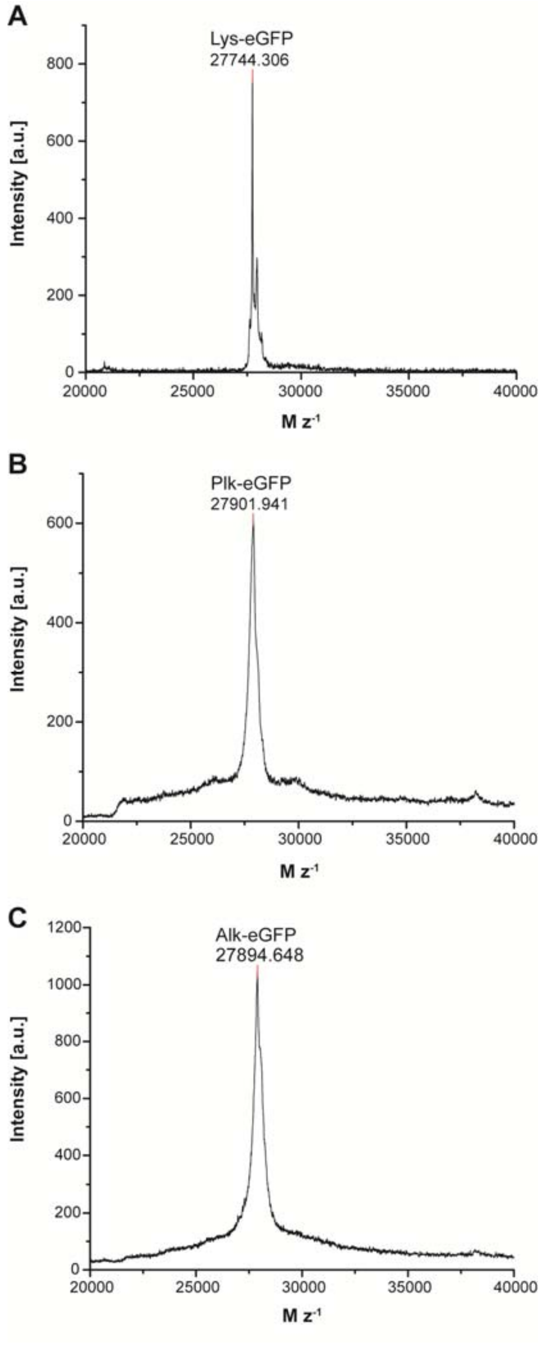

Figure S1. **MALDI-MS analysis of eGFP analogues.** (A) Lys-eGFP (obs. average mass 27744.306 Da, calc. average mass 27745.17 Da). (B) Plk-eGFP Obs. average mass 27901.941 Da, calc. average mass 27827.24 Da. (C) Alk-eGFP Obs. average mass 27894.648 Da, calc. average mass 27858.24 Da.

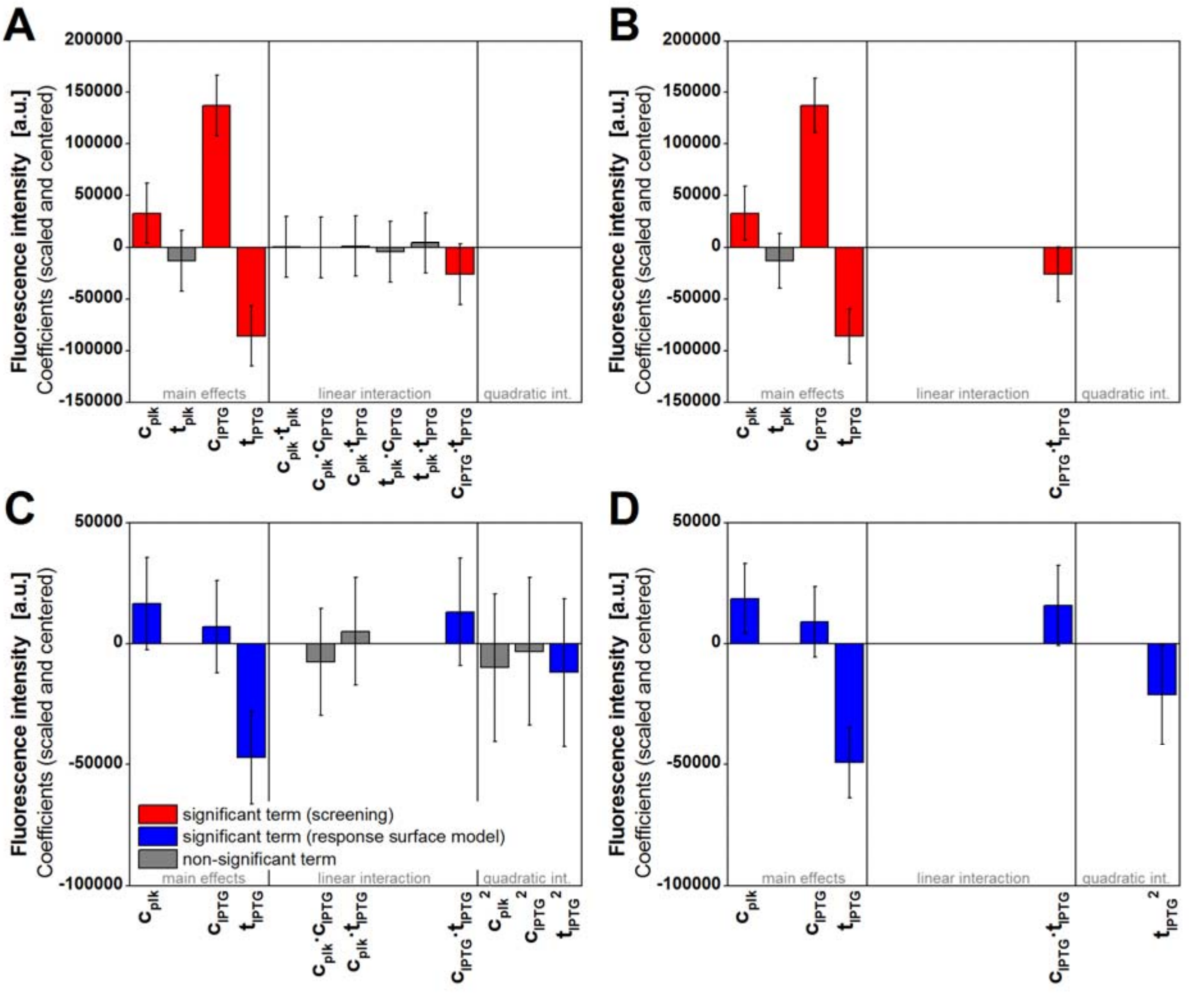

Figure S2. Significance of model coefficients. Each bar represents the impact of a regression coefficient on the fluorescence intensity when a factor varies from its maximum value to its minimum value (parameter ranges in **Table S1**). The coefficients were scaled and centered for comparison. In this presentation a coefficient is significant when the errors bars intersect the abscissa. (A, B) Coefficients for the screening model and (C, D) the response surface model are presented. Based on the selected experimental design 10 terms (main: Plk concentration c_{plk}, Plk time of addition t_{plk}, IPTG concentration c_{IPTG}, IPTG time of addition t_{IPTG}; linear interactions: $c_{plk} \cdot t_{plk}$, $c_{plk} \cdot c_{IPTG}$, $c_{plk} \cdot t_{IPTG}$, $t_{plk} \cdot c_{IPTG}$, $t_{plk} \cdot t_{IPTG}$, $c_{IPTG} \cdot t_{IPTG}$) were estimated (A) and 5 (main: c_{plk}, t_{plk}, c_{IPTG}, t_{IPTG}; linear interactions: $c_{IPTG} \cdot t_{IPTG}$) were used for modelling the screening design

space (B). With respect to the experimental design, the time point when plk was added to the cultivation broth (t_{plk}) was kept as part of the screening model although this factor was non-significant. Based on the results of the screening model 9 terms (main: c_{plk}, c_{IPTG}, t_{IPTG}; linear interactions: $c_{plk} \cdot c_{IPTG}$, $c_{plk} \cdot t_{IPTG}$, $c_{IPTG} \cdot t_{IPTG}$; quadratic interactions: c_{plk}^2, c_{IPTG}^2, t_{IPTG}^2) were addressed for the response surface model (C) of which 5 were significant (main: c_{plk}, c_{IPTG}, t_{IPTG}; linear interactions: $c_{IPTG} \cdot t_{IPTG}$; quadratic interaction: t_{IPTG}^2) and used for the estimation of the robust set point (D).

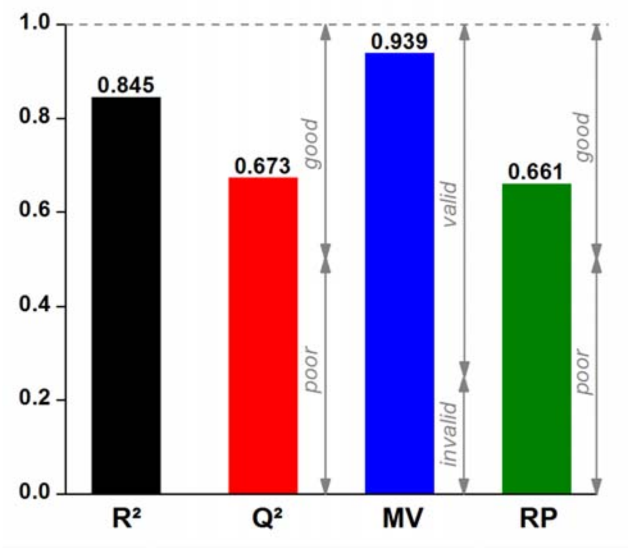

Figure S3. Evaluation of the response surface model according to standard design of experiments procedures[3]. The goodness of fit (R^2) and goodness of prediction (Q^2) indicate that the constructed response surface model adequately predicted useful point of operation for the production of Plk-eGFP ($Q^2 > 0.5$, $R^2 - Q^2 < 0.2$). As shown by the model validity (MV) the response surface model with quadratic interactions was appropriate (MV > 0.25) and the experimental reproducibility (RP) can be adequately controlled (RP > 0.5) .

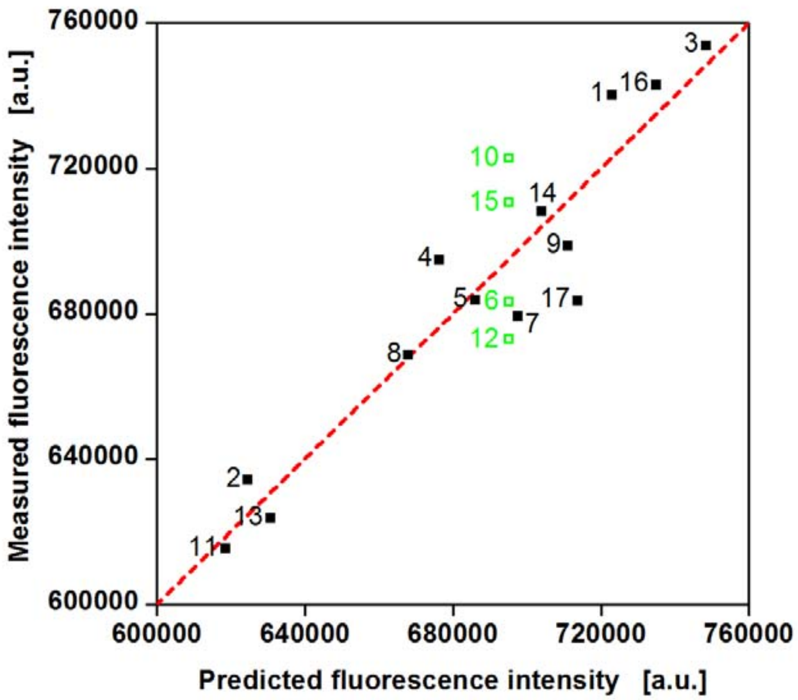

Figure S4. Measured and predicted eGFP fluorescence intensities for the response surface model validation run were compared. The run order is given by the labels and the red dashed line indicates the bisecting line where the predicted values are equal to the measured values. The predicted fluorescence intensities were in good accordance with the measured values. Cultivations 6, 10, 12 and 15 (green) were center points where each factor is set to the mean setting of the experimental design (**Figure S2**). The deviations between the repeated cultivations (center points) indicated the scattering of the measurement system and were compared with the overall variation in the experimental space (**Figure S3**).

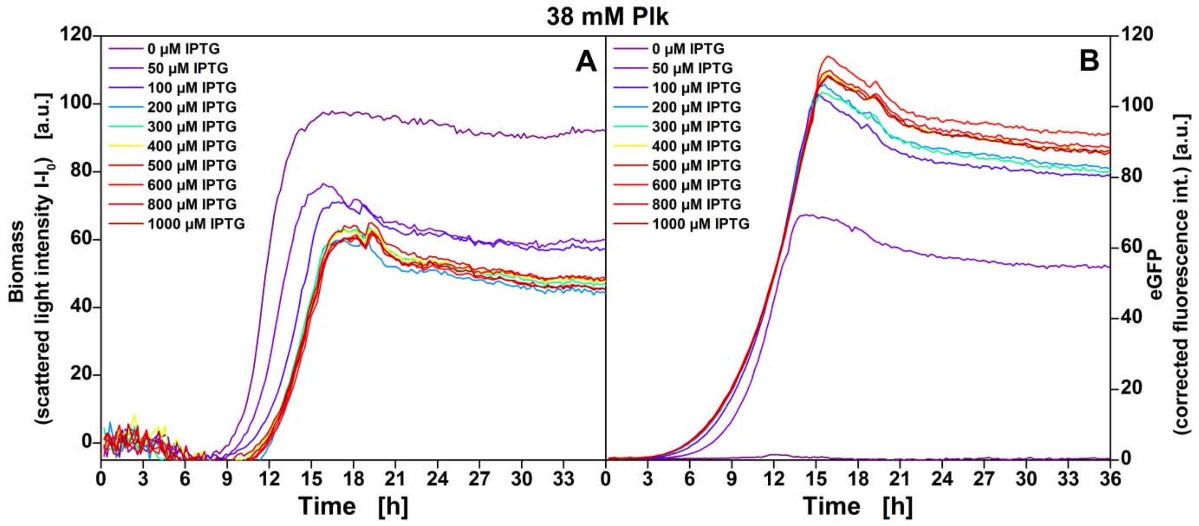

Figure S5. Variation of IPTG concentration. Since the constructed response surface model predicted slightly higher fluorescence intensity for an IPTG concentration of 300 µM instead of 433 µM at the set point (**Figure 5 and Figure S6**) an IPTG concentration gradient was investigated to validate the set point (c_{IPTG} = 0 - 1000 µM). The scattered light intensity monitoring showed delayed and lower growth due to metabolic burden of heterologous protein production for IPTG concentrations of 0 – 200 µM and saturated induction for IPTG concentrations of 200 – 1000 µM IPTG (A). Plk-eGFP fluorescence strongly increased between 0 and 100 µM IPTG (B). At 200 and 300 µM IPTG the final eGFP fluorescence after 36 h was about 8 % lower than for 400 – 1000 µM IPTG where the final Plk-eGFP fluorescence intensities were maximal and within ± 3 %. The IPTG concentration of 433 µM at the robust set point was, therefore, suited to achieve maxium Plk-eGFP concentration. Cultivation conditions: 780 µL Wilms-MOPS mineral medium per well in 48-FlowerPlates, $OD_{600,t=0}$ = 0.1, 30 °C, shaking frequency: 1000 rpm, shaking diameter: 3 mm, c_{plk} = 38 mM, t_{plk} = 0 h, t_{IPTG} = 0 h.

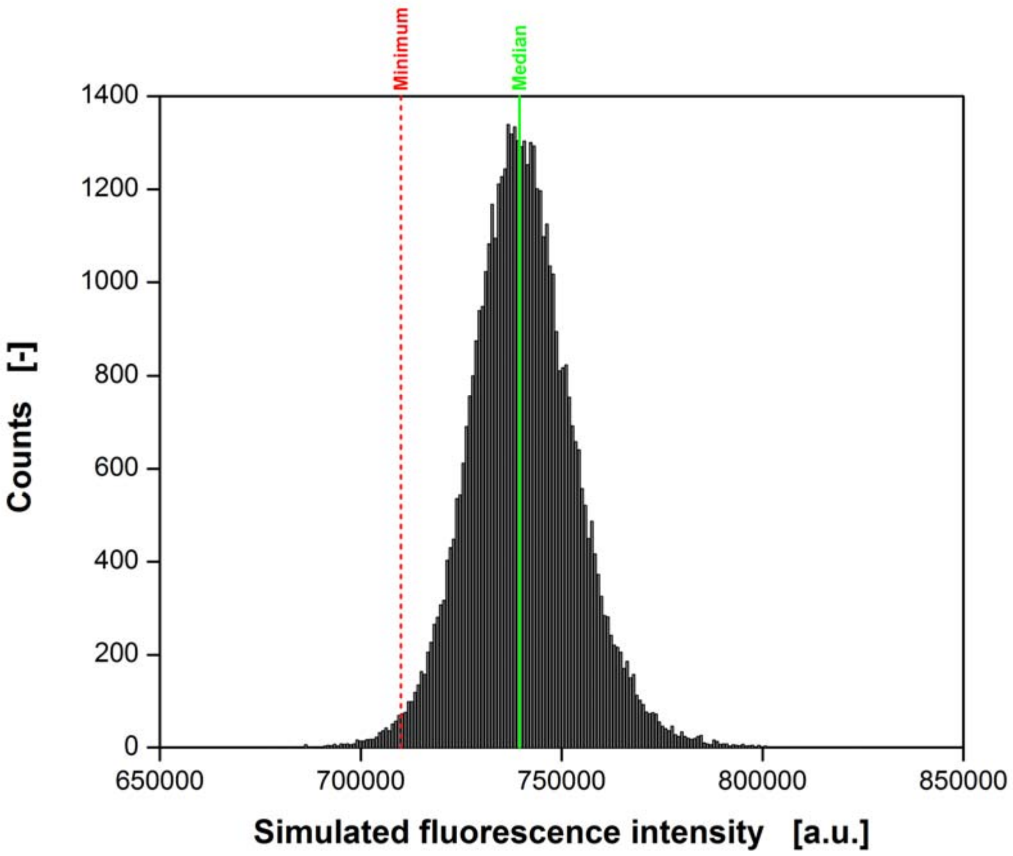

Figure S6. Determination of the operating point via Monte-Carlo simulation. Based on the results of the response surface model, a robust set point for the production of Plk-eGFP was found. A minimum fluorescence intensity of 710000 a.u. (red dashed line) was defined as requirement of the robust set point. 50000 fluorescence intensity predictions were performed with random variations of the factor setting within the confidence interval of each factor. The green line indicates the median of the simulated fluorescence intensities. Since the median of the simulated fluorescence intensities was above the required minimum fluorescence intensity, the selected robust set point fulfilled the requirements. The process parameters at the robust set point were: c_{plk} = 38 mM, t_{plk} = 0 h, c_{IPTG} = 433 µM, t_{IPTG} = 0.2 h. Out of practicability IPTG was added at the start of the cultivation (t_{IPTG} = 0 h) in further experiments (and not at 0.2 h).

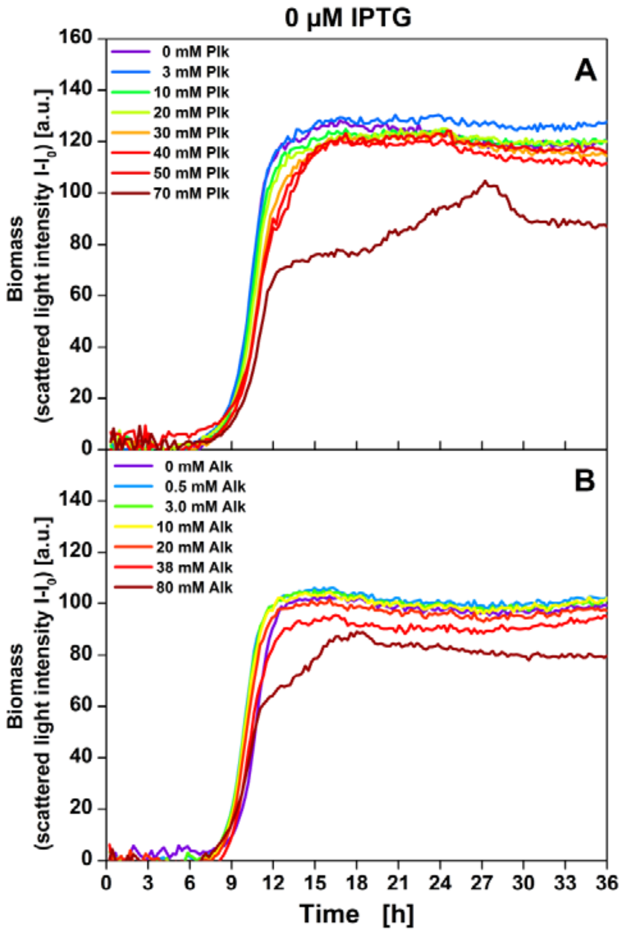

Figure S7. Effect of high Plk and Alk concentrations. Since high uAA concentrations correlated with uAA-eGFP formation (**Figure 6**) the influence of high uAA concentrations on the growth of non-induced cultures (0 µM IPTG) was investigated. For example, Plk or Alk might be metabolized as additional substrate leading to additional growth or have toxic effects leading to reduced growth rates.(A) Plk in concentrations of up to 50 mM caused minor deviations in the scattered light signal. A slight retardation was observed in the late growth phase for increasing concentrations (11 -14 h). At a very high concentration of 70 mM a strong deviation in growth was observed and the final scattered light intensity was reduced by 25 %. (B) For Alk no changes were observed up to 20 mM. The final scattered light intensity of a cultivation with 80 mM Plk was reduced by 22 %. In summary, for uAA concentration that lead to high uAA-eGFP

concentrations (i.e. 38 mM Plk, 10 mM Alk) no considerable effects on the growth of non-induced cultures was observed and the high uAA-EGFP concentrations were, therefore, not a result of metabolization of the uAA as additional substrate. Cultivation conditions: 780 µL Wilms-MOPS mineral medium per well in 48-FlowerPlates, $OD_{600,t=0}$ = 0.1, 30 °C, shaking frequency: 1000 rpm, shaking diameter: 3 mm, t_{uAA} = 0 h, t_{IPTG} = 0 h.

References:

1. Zimmermann T, Rietdorf J, Pepperkok R: Spectral imaging and its applications in live cell microscopy. *Febs Letters* 2003, **546:**87-92.
2. Lichten CA, White R, Clark IBN, Swain PS: Unmixing of fluorescence spectra to resolve quantitative time-series measurements of gene expression in plate readers. *Bmc Biotechnology* 2014, **14**.
3. Eriksson L, Johansson E, Kettaneh-Wold N, Wikström C, Wold S: *Design of Experiments: Principles and Applications. Third revised and enlarged edition.* Umeå, Sweden: MKS Umetrics AB; 2008.
